# Patched Homolog 1 (PTCH1) Mutation in a CIC-Rearranged Sarcoma: Lack of Response to the Smoothened (SMO) Vismodegib

**DOI:** 10.7759/cureus.34281

**Published:** 2023-01-27

**Authors:** David J Wu, Paari Murugan, Keith M Skubitz

**Affiliations:** 1 Medicine, University of Minnesota, Minneapolis, USA; 2 Laboratory Medicine and Pathology, University of Minnesota, Minneapolis, USA

**Keywords:** ewing sarcoma family of tumors (esft), sarcoma, personalized medicine, tyrosine kinase inhibitor, next-generation sequencing, hedgehog, vismodegib, ewing, cic-rearranged sarcoma, ptch1

## Abstract

Next-generation sequencing (NGS) to identify potential targets is becoming a common approach to refractory tumors. We describe a patient with a CIC-DUX4 sarcoma that harbored a patched homolog 1 (PTCH1) mutation, a mutation not previously reported in so-called Ewing family tumors. PTCH1 is part of the hedgehog signaling pathway. Basal cell carcinomas (BCC) commonly have PTCH1 mutations, and those with PTCH1 mutations are often responsive to therapy with the hedgehog pathway inhibitor vismodegib. The effect of any mutation in a gene important in cell growth and division is likely dependent upon the background biochemistry of the cell. In the current case, vismodegib was not effective. This case is the first report of a PTCH1 mutation in an Ewing family tumor and demonstrates that the utility of targeting a potential mutation may depend upon many factors, including other mutations in the signaling pathway, and importantly, also the background biochemistry of the malignant cell that may prevent effective treatment targeting.

## Introduction

The dramatic response of a gastrointestinal stromal tumor (GIST), a tumor for which no effective therapy was previously available, to the KIT tyrosine kinase inhibitor (TKI) imatinib [1] represented the first demonstration of the potential of small molecule TKI inhibitors in solid tumors. For some cancers, targeted therapy now shows a higher response rate and longer survival than conventional chemotherapy, and currently targeted therapy is the preferred approach for some tumors such as lung adenocarcinoma with specific underlying mutations [2]. While some tumors such as GISTs and certain lung cancers are routinely characterized by mutations responsive to a TKI, most tumors do not have currently well-defined targets for such therapy. In the current era of personalized medicine, the use of next-generation sequencing (NGS) to identify potential targets is becoming more prevalent. Nevertheless, the effect of any mutation in a gene important in cell growth and division is likely highly dependent upon the background biochemistry of the cell. Therefore, the identification of a potential target does not necessarily guarantee the efficacy of a drug that inhibits that target.

Soft tissue sarcomas (STS) can be grouped into two broad categories: those with specific cytogenetic changes and generally relatively simple karyotypes, and those with more varied non-specific mutations and often complex genetic changes. The small round cell sarcomas previously termed the Ewing family of tumors are generally categorized by specific chromosomal translocations and relatively few other mutations [3]. We describe a case of an undifferentiated small round cell sarcoma with a characteristic CIC fusion, which was previously classified as an Ewing family tumor, who relapsed following standard chemotherapy. CIC-DUX4 sarcomas have been shown to have a wide spectrum of morphology with generally a more aggressive course and inferior overall survival as compared to Ewing sarcoma [4]. NGS in our patient revealed a mutation in the cell surface transmembrane receptor patched homolog 1 (PTCH1).

PTCH1 is part of the sonic hedgehog (SHH) signaling pathway [5]. The SHH signaling pathway is initiated by the cell surface receptor smoothened homolog (SMO), which is normally inhibited by PTCH1. The binding of the SHH ligand to PTCH1 prevents this inhibition and allows downstream SHH signaling to proceed. If SMO is not inhibited by PTCH1, it can interact with the suppressor of fused homolog (SUFU), which regulates the activation of the glioma-associated oncogene homologs (GLI) family of transcription factors [6]; thus, SMO signaling activates GLI.

Mutations in the SHH pathway have not been reported in Ewing family tumors; however, they have been implicated in cancers such as nevoid basal cell carcinoma (BCC) syndrome, also known as Gorlin-Goltz syndrome, a rare autosomal dominant, tumor-predisposing disorder caused by germline pathogenic variants in the human homolog of the PTCH1 gene [7]. In other forms of BCC, two different mechanisms have been shown to be pathogenic: mutations in PTCH1 can prevent inhibition of SMO activation of the SHH pathway [8], or mutations of SMO can result in constitutive activation of the pathway [9].

Vismodegib is a small molecule inhibitor of SMO and was approved by the Food and Drug Administration (FDA) for the treatment of locally advanced and metastatic BCC in 2012 [9]. BCCs commonly have PTCH1 mutations, and those with PTCH1 mutations are often responsive to therapy with vismodegib [10]. We, therefore, treated this patient with vismodegib, but the tumor rapidly progressed. This case is the first report of a PTCH1 mutation in a so-called Ewing family tumor and demonstrates that the utility of targeting a potential mutation may depend upon many factors such as the background biochemistry of the malignant cell that may prevent effective treatment targeting.

## Case presentation

A 36-year-old woman had a growing nodule removed from her forearm. Two nodules recurred at the operative site two months later that was removed; examination revealed a sarcoma. Pathology review was consistent with CIC-rearranged primitive round cell sarcoma. One month later another nodule appeared at the operative site.

**Figure 1:**
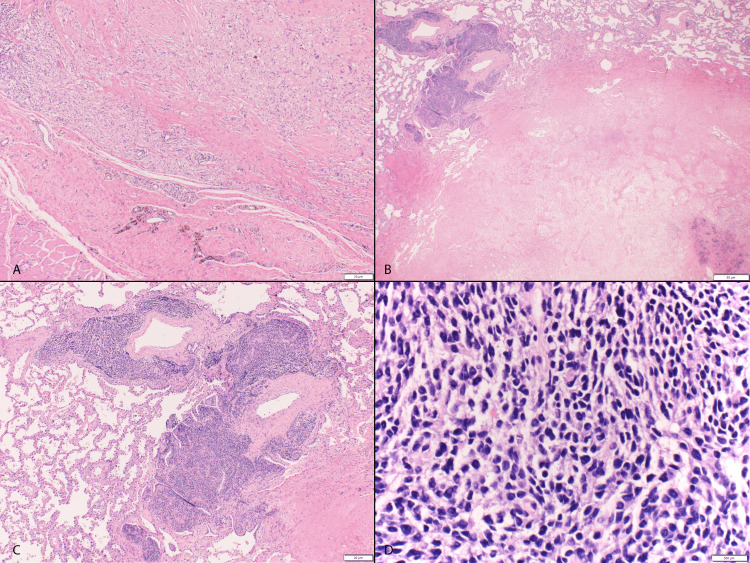
Resected forearm nodule status post-CAV/IMV chemotherapy Resected forearm nodule status post cyclophosphamide, doxorubicin, and vincristine (CAV) and ifosfamide and etoposide (IMV) chemotherapy demonstrating subcutaneous tumor bed with therapy-related changes including fibrosis, histiocytic infiltration, and hemosiderin deposition and lack of residual sarcoma (A, hematoxylin and eosin (H&E) X40). Metastatic lung nodule with focal viable tumor (upper left) and extensive necrosis (B, H&E X20). Viable sarcoma demonstrating sheets of small round blue cells clustered around pulmonary vessels (C, H&E X40). Tumor cells with hyperchromatic round to oval nuclei, inconspicuous nucleoli, and moderate amounts of clear to pale eosinophilic cytoplasm in a myxoid background, consistent with CIC-rearranged sarcoma (D, H&E X400).

She started cyclophosphamide, doxorubicin, and vincristine (CAV) alternating with ifosfamide and etoposide (IMV). After three cycles of each and a discussion at the multidisciplinary tumor board, she had the tumor removed due to concern about a lack of further tumor shrinkage. The pathology on the resection showed no definite residual viable tumor (Figure 1). She had three more cycles of CAV and one more IMV after surgery and experienced prominent myelosuppression, limiting further chemotherapy. Computed tomography (CT) imaging surveillance was performed every three months. Twenty-six months after starting chemotherapy, a 1-cm lung nodule was noted and removed; pathological examination showed a malignant round cell neoplasm with 90% necrosis consistent with metastasis of the CIC-rearranged sarcoma (Figure 1). One month later she developed visual changes and was found to have brain metastases along with liver metastasis and mediastinal lymphadenopathy. NGS using the FoundationOne platform on the original specimen showed a PTCH1 A1380V mutation along with the CIC-DUX4 fusion. FoundationOne sequencing on the lung nodule showed a PTCH1 A1380V mutation and 1 Mut/Mb, with no detectable change compared with the original tumor.

She was treated with stereotactic radiosurgery (GammaKnife, Leksell) to the brain lesions and began treatment with pegylated liposomal doxorubicin (PLD). PLD was chosen due to the potential for activity and quality-of-life considerations. Her performance status improved, and imaging showed a response of the liver lesion one month later. This response was maintained for another four months until her imaging showed tumor progression.

The NGS on her original tumor and subsequent lung sample both had a mutation of PTCH1 A1380V, suggesting potential vismodegib sensitivity. She began vismodegib at 150 mg/d. One month later her performance status was worse, and imaging showed the progression of the disease. She then started cyclophosphamide and topotecan and discontinued vismodegib. Imaging five weeks later showed progressive disease in the chest and new brain lesions. Pazopanib was begun at 800 mg/d, but imaging showed progression of the disease four weeks later. Hospice was discussed, but she preferred to try high-dose ifosfamide; the tumor progressed rapidly after one cycle, and she enrolled in hospice three weeks later.

## Discussion

In our patient with metastatic CIC-rearranged sarcoma unresponsive to standard therapy, NGS identified a mutation in the PTCH1 gene, the receptor for an SHH that is involved in SHH signaling. PTCH1 mutations have been found in BCC and osteosarcoma [11,12], but have not been reported in Ewing family tumors. Vismodegib, an inhibitor of SMO and the SHH pathway, is now standard treatment in metastatic BCC or locally advanced BCC not amenable to resection or radiotherapy [10]. The tumor in our case did not respond to vismodegib.

**Figure 2:**
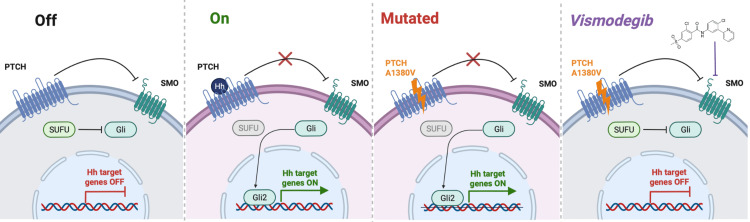
Sonic hedgehog pathway with the mechanism of PTCH mutation and vismodegib activity Sonic hedgehog pathway with the mechanism of patched homolog (PTCH) mutation and vismodegib activity. Normally, PTCH inhibits smoothened homolog (SMO) and downstream expression of Hedgehog (Hh) genes is turned off. The binding of Hh to PTCH causes inhibition of SMO, leading to activation of glioma-associated oncogene homologs (GLI) and expression of Hh genes. The PTCH A1380V mutation disables PTCH inhibition of SMO, leading to unregulated Hh gene expression. Vismodegib is a small-molecule inhibitor that is able to bind to SMO and turn off GLI activation, leading to attenuation of Hh expression. Figure produced in BioRender by DJW. SUFU: suppressor of fused homolog

The SHH pathway was first identified in drosophila and is critical for both normal embryonic development and tissue homeostasis in adult skin, hair, and other cells [13]. PTCH1, a 12-pass transmembrane protein, is a receptor for hedgehog ligands Sonic, Indin, and Desert. PTCH1 normally represses SMO, a 7-pass transmembrane G protein-coupled receptor protein, and binding of ligands to PTCH1 removes this inhibition (Figure 2). SMO interacts with SUFU, which regulates the activation of the GLI family of transcription factors; thus, SMO signaling activates GLI [6,13]. Vismodegib, an SMO inhibitor, has been found to have significant activity in BCC and medulloblastoma, with objective response rates of 65% in locally advanced BCC and 37% in medulloblastoma [14,15]. Alterations that inactivate PTCH1 may also predict sensitivity to vismodegib in BCC and medulloblastoma [16,17]. There is little data on the use of vismodegib in sarcoma, with no retrospective or prospective data yet available.

There are several possible reasons for the lack of anti-tumor response to vismodegib in our patient. Treatment-resistant mutations to vismodegib have been reported, with the vast majority of vismodegib resistance in PTCH1 mutation-driven BCC caused by acquired mutations in the drug target, SMO. NGS in our patient did not reveal any SMO mutations. A few cases have reported vismodegib resistance in patients with PTCH1 mutations [18,19]. Intra-tumor heterogeneity has also been shown to correlate with vismodegib resistance [20].

Another possible mechanism of resistance is that the background biochemistry of our patient’s sarcoma is different from tumors such as BCC, such that the cells are much less dependent on SHH signaling. Thus, our patient’s CIC-DUX4 rearrangement, resulting in a fusion oncoprotein comprising the transcriptional repressor CIC fused to the C-terminal transcriptional activation domain of DUX4 [4] as the main driver of the disease may override the effects of SHH signaling in our case. There are no therapies targeting CIC-DUX4 rearrangements available at present.

Pazopanib was tried after the tumor progressed on vismodegib. Pazopanib is a multitarget TKI that inhibits vascular endothelial growth factor (VEGF) and other tyrosine kinases and is approved by the FDA for the treatment of patients with advanced STS. Interestingly, case studies have shown that pazopanib can inhibit cell proliferation and PDGFRβ phosphorylation in GLI1-overexpressed cells, suggesting it may have an inhibitory effect on the SHH pathway as well [17].

In summary, we report a case of CIC-rearranged sarcoma with a PTCH1 mutation; however, the targeted therapy chosen from the NGS results was not effective. Targeted therapy remains a promising area of cancer research, but its success may depend on the background biochemistry of the tumor cell under study, in addition to specific resistance mutations in the signaling pathway targeted. In addition, our case provides further evidence of the potential utility of PLD in “refractory” Ewing family tumors given its low degree of myelosuppression and general tolerability in this typically heavily pretreated group of patients.

## Conclusions

We describe a patient with a CIC-DUX4 sarcoma that harbored a PTCH1 mutation, a mutation not previously reported in so-called Ewing family tumors. Although NGS may be useful to identify potential targets for cancer therapy, the biological effect of a mutation in a gene important in cell growth and division is likely dependent upon the background biochemistry of the cell. While BCC with PTCH1 mutations are often responsive to therapy with the hedgehog pathway inhibitor vismodegib, this agent was not effective in the current patient. This case demonstrates that the utility of targeting a potential mutation may depend upon many factors such as the background biochemistry of the malignant cell that may prevent effective treatment targeting.
